# Neighboring Gulf Killifish (*Fundulus grandis*) Populations Differ in Their Plastic and Genetic Responses to Salinity Fluctuations

**DOI:** 10.1002/ece3.74115

**Published:** 2026-08-07

**Authors:** Antrelle D. Clark, Adam Hallaj, Christopher J. Anderson, Moisés A. Bernal

**Affiliations:** ^1^ Department of Biological Sciences Auburn University Auburn Alabama USA; ^2^ College of Forestry, Wildlife and Environment Auburn University Auburn Alabama USA; ^3^ Smithsonian Tropical Research Institute Panama Panama

**Keywords:** coastal urbanization, environmental fluctuations, gill, killifish, osmotic stress, salinity

## Abstract

Coastal development is increasing rapidly, intensifying the frequency and magnitude of watershed runoff and salinity fluctuations in estuarine ecosystems. In developed areas, impervious surfaces can accelerate freshwater runoff during rains, producing rapid and extreme osmotic shifts that challenge coastal organisms. Along the northern Gulf of Mexico (GoM), the Gulf killifish ( *Fundulus grandis* ) is a species that is consistently present in reference and urbanized tidal creeks. To evaluate responses to osmotic fluctuations, we conducted a laboratory‐based reciprocal transplant experiment using individuals from a reference watershed (Long Bayou, AL) and an urbanized watershed (Weakley Bayou, FL) along the northern GoM. Individuals from each population were exposed to either a simulated tidal event (low fluctuation; 15–10 ppt) or a tide/runoff event (high fluctuation; 15 ppt to 10 ppt to 5 ppt) over an acute period (~7 h) to assess responses via gill gene expression. RNA‐seq analyses identified 112 differentially expressed genes (DEGs), and principal component analysis showed separation by salinity treatment along PC1 (31% variance) and separation by population origin along PC2 (13% variance). The urban population showed a substantially higher number of DEGs compared to the reference population. Processes primarily activated in the urban population under osmotic stress were associated with ion transport, negative regulation of cell cycle, cellular integrity, and energy reallocation. Transcriptome‐derived single nucleotide polymorphisms (SNPs) revealed small but significant genetic differentiation (*F*_ST_ = 0.021, *p* < 0.05), with higher nucleotide diversity and lower inbreeding in the urban population (*π* = 0.106; FIS = 0.004). This study indicates that both adaptation and plasticity can shape responses to abiotic variability in coastal environments at small spatial scales.

## Graphical abstract

Coastal development can alter the salinity fluctuations experienced by tidal creek systems. Understanding how resident organisms, such as the Gulf killifish, respond to these changes is critical, as development along the coastline is projected to triple in the coming decades.

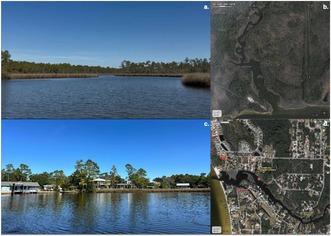

## 1 Introduction

Coastal ecosystems are highly dynamic environments in which abiotic conditions (e.g., salinity, flooding, dissolved oxygen, temperature, etc.) fluctuate regularly. While natural fluctuations already challenge marine organisms from a physiological standpoint, the magnitude and frequency of abiotic changes can be accentuated by urbanization. Globally, urban land use has expanded over recent decades and is projected to increase substantially by the year 2050, particularly along coastal regions (Nowak and Walton 2005). This expansion alters water quality and drainage patterns, as urban land use is strongly associated with increased stormwater runoff and negative impacts on ecosystem health (Walsh et al. 2005; Wedge et al. 2015; Levin et al. 2020).

For instance, a coastal ecosystem currently being affected by urban development are tidal creeks in the northern Gulf of Mexico (GoM). Impervious surfaces such as roads, roofs, and sidewalks limit freshwater infiltration into the soil during intense storms (Paul and Meyer 2001; Holland et al. 2004; Kültz 2015; Sahoo and Smith 2009). The resulting freshwater runoff can cause rapid decreases in salinity, posing physiological challenges for organisms in tidal creeks (Min et al. 2011; Sanger et al. 2015; Komoroske et al. 2016; Noori et al. 2016). These urban‐driven fluctuations have a direct impact on salt marsh communities, altering seasonal patterns of fish diversity and abundance across reference and urbanized GoM sites (Wedge and Anderson 2017). Previous studies have shown that even small changes in coastal land cover can alter salinity patterns, imposing higher physiological demands on resident organisms beyond those experienced under natural conditions (Bickley and Anderson 2025). Despite these challenges, the Gulf killifish ( *Fundulus grandis* ) is the only fish species consistently present in all tidal creeks of the northern GoM, regardless of the magnitude or frequency of osmotic fluctuations (Wedge and Anderson 2017). It is also understood that *F. grandis* exhibits high site fidelity and does not move extensively from their initial range (Nelson et al. 2014). These factors make the Gulf killifish a well‐suited candidate for studying responses of coastal species to different salinity regimes at small spatial scales across tidal creeks exhibiting variation in environmental conditions associated with land cover differences.

In response to such intensified environmental stressors, resident organisms may rely on phenotypic plasticity, where shifts in molecular and physiological processes allow individuals to better withstand acute environmental variations (Gotthard and Nylin 1995). In addition, populations may exhibit local adaptation, in which genetic differences accumulate among populations to enhance performance under specific abiotic conditions (Ghalambor et al. 2007; Fox et al. 2019). Killifish in the genus *Fundulus* are well known for their ability to tolerate extreme fluctuations in environmental parameters (Griffith 1974; Whitehead 2010). Previous studies on the Atlantic killifish (also known as the mummichog, *F. heteroclitus* ) suggest that they can efficiently acclimate through transcriptional regulation, which mediates gill restructuring to match the surrounding environment and maintain osmotic balance (Whitehead, Galvez, et al. 2011; Whitehead et al. 2012). Additionally, salinity can drive genetic divergence between populations, leading to local adaptation in the Atlantic killifish (Whitehead, Roach, et al. 2011). Despite these advances, questions remain on the potential for local adaptation and phenotypic plasticity in the closely related Gulf killifish ( *F. grandis* ) to different levels of salinity fluctuation, promoted in part by coastal urbanization. Findings from Waldo et al. (2023) suggest that genetic divergence between populations is associated with geographic distance and differences in salinity regimes in the northern GoM. However, it remains unclear if populations in urbanized sites respond differently to salinity fluctuations when compared to reference sites. Further, questions remain on whether the Gulf killifish primarily rely on transcriptional acclimation to cope with osmotic stress or whether local adaptation contributes to salinity tolerance on sites with different salinity regimes.

The Gulf killifish is abundant and widely distributed across tidal creeks and salt marshes in the northern GoM, where it frequently experiences natural and anthropogenically amplified salinity fluctuations. Its role as an intermediate species and indicator of overall ecosystem stability (Vivian et al. 2012; Wedge and Anderson 2017; Lu et al. 2018), coupled with the well‐documented tolerance of *Fundulus* species to extreme environmental fluctuations (Crego and Peterson 1997; Whitehead 2010; Whitehead, Galvez, et al. 2011; Whitehead, Roach, et al. 2011), make it a good candidate for examining plasticity and potential local adaptation in response to changing environments.

In this study, we conducted a laboratory‐based reciprocal transplant experiment with two populations of *F. grandis* with prior exposure to contrasting environmental conditions associated with coastal urbanization: a reference site with low salinity fluctuation and an urbanized site with high fluctuation. Although determining the effects of urbanization requires replication across multiple urban and reference sites to distinguish those effects from site‐specific variation, single‐site comparisons such as the present study can identify population‐level differences associated with urbanized environments and provide a basis for testing whether these patterns are consistent across broader spatial scales (Marshall 2024). The three main questions addressed in this study were: (1) Do Gulf killifish respond differently to acute salinity fluctuations based on population origin, as measured by gill differential gene expression? (2) What are the molecular processes associated with fast salinity fluctuations in tidal streams for the killifish? and (3) Are the differences in response to salinity fluctuations associated with genetic divergence between individuals from the two sites? We hypothesized that both population origin and salinity treatment would significantly influence gill gene expression in *F. grandis* , with population origin having the greatest impact. We also predicted that genetic differentiation between the two populations would contribute to differences in osmotic regulation, suggesting potential local adaptation in the Gulf killifish. This study highlights the importance of evaluating species' responses to environmental stressors across multiple populations, particularly in areas where urbanization is quickly expanding.

## 2 Methods

### 2.1 Site Description and Fish Collection

**FIGURE 1:**
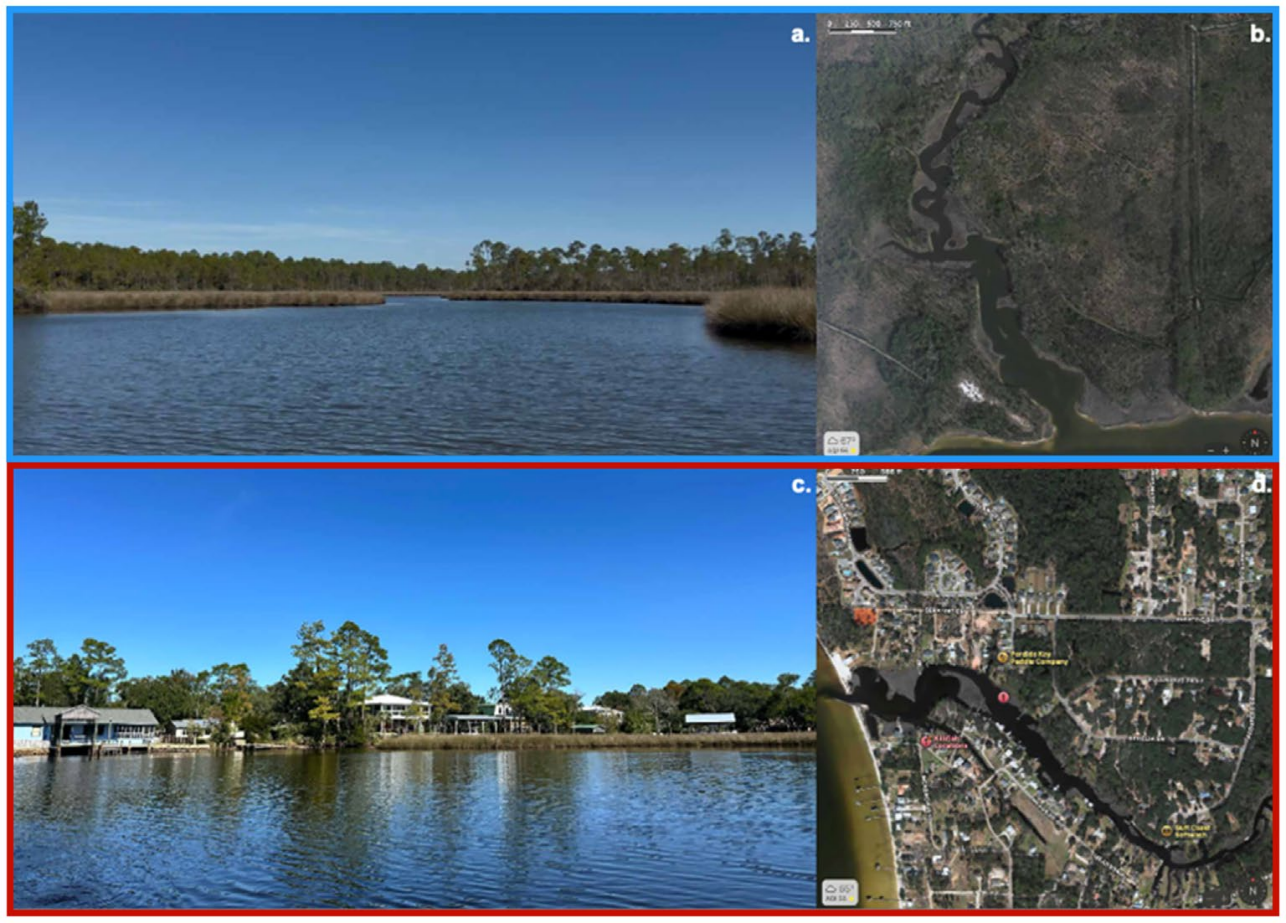
In‐field and aerial views of each study area. (a, b) Reference site: Long Bayou. (c, d) Urban site: Weakley Bayou. (Images from Google maps, 2025).

For this study, fish were collected at two tidal creek salt marsh sites in the Perdido‐Wolf Bay system located along the boundary of Alabama and Florida, USA. Creeks sampled were Long Bayou (LB; 30.30909, −87.6236), representing a reference site (Figure 1a,b), and Weakley Bayou (WB; 30.35208, −87.4118), representing an urban site (Figure 1c,d; Waldo et al. 2023; Bickley and Anderson 2025). Sites were selected because of recent research and background information collected at the creeks and the presence of *F. grandis* (Wedge and Anderson 2017; Bickley et al. 2024; Bickley and Anderson 2025). Based on the 2016 National Land Cover Dataset (Dewitz 2019), the drainage area of LB was 6.8 km^2^ with 3.3% classified as urban land cover, while WB had a similar drainage area (6.7 km^2^) but a higher proportion categorized as urban (17.6%), most of which was concentrated near or along the creek (Bickley and Anderson 2025). Surface water salinity variability has been monitored at both creeks in two separate studies. Salinity measures at WB and two other urban creeks were evaluated between April 2012 and March 2013 and showed frequent rapid fluctuations and low salinity measures (< 5 ppt) 5.9% of the time, compared to LB which was only 0.9% (Wedge and Anderson 2017). Another study calculated average hourly changes in salinity (mean salinity time step change, MSTΔ), and seasonal averages for MSTΔ ranged between 0.15–0.17 ppt for WB and 0.04 ppt for LB (Bickley and Anderson 2025). These differences indicate that individuals in the urban site WB experience greater salinity fluctuations than the reference site LB.

Fish were collected at each creek in October 2020 using basket traps (22.9 cm × 44.5 cm, with a 2.5‐cm opening on each end) baited with commercially available bait (*Brevoortia* sp. and *Selar crumenophthalmus* ; Waldo et al. 2023). Traps were deployed during high tide and retrieved 4 h later. Forty‐eight adult individuals of *F. grandis* (average length: 8.39 ± 1.81 cm; average weight: 12.66 ± 7.64 g; sex: undetermined) were transported in aerated buckets to Auburn University facilities (Auburn, AL, USA), under Auburn University's approved Institutional Animal Care and Use Committee (IACUC) permit #2018‐3396. Fish were acclimated to a temperature of 25°C ± 1°C and a salinity of 15 ± 1 parts per thousand (ppt) for 8 days. All fish were fed tropical fish pellets (size small; Ocean Nutrition Formula One Pellets) twice a day throughout the acclimation period and on the morning of the salinity exposure experiment. The fish were grouped by origin and housed in sets of six individuals per tank. The aquarium tanks were of 75 L (20‐gal), equipped with a Finnex aquarium heater to maintain temperature, a Fluval AquaClear 30 Power Filter, and a Nicrew ClassicLED Plus Aquarium light set on a 12:12 light:dark cycle.

### 2.2 Experimental Approaches

**FIGURE 2:**
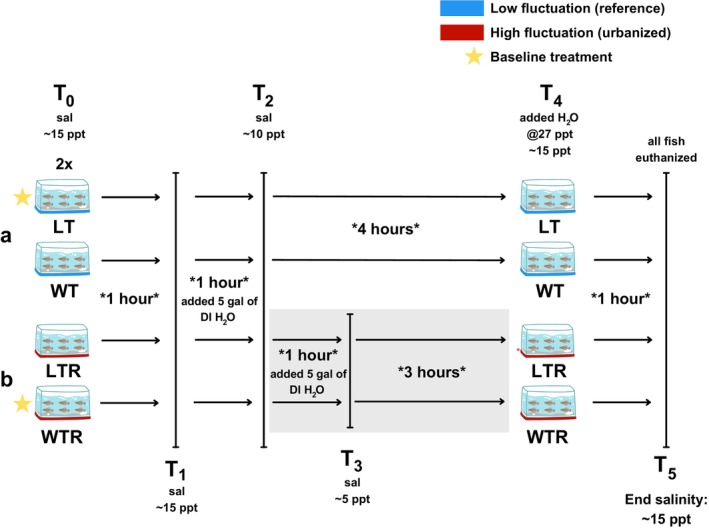
Reciprocal transplant experimental design to assess differences in response to acute osmotic stress based on population origin. Each treatment began with a 1‐h acclimation period at 15 ppt and ended with a 1‐h recovery period after salinity was returned to 15 ppt. (a) Tide treatment (low fluctuation; LT/WT): Individuals from both populations started at 15 ppt, were dropped to 10 ppt for 4 h, then returned to 15 ppt. (b) Tide/Runoff treatment (high fluctuation; WTR/LTR): Individuals from both populations started at 15 ppt, were dropped to 10 ppt for 1 h before being further reduced to 5 ppt for 3 h, then returned to 15 ppt. Stars indicate the population's baseline treatment.

Gulf killifish were acclimated to aquarium conditions for 8 days prior to the start of the experiment. The experimental design consisted of two low fluctuation groups (Long Tide = LT, Weakley Tide = WT) and two high fluctuation groups (Long Tide/Runoff = LTR, Weakley Tide/Runoff = WTR). Each one of the treatments was replicated in two separate tank systems for each origin of population (*n* = 24 per population; *n* = 12 per treatment; *n* = 6 per tank). The experiment lasted 7 h and consisted of a 1‐h acclimation period at 15 ppt prior to the respective salinity treatment, and a 1‐h recovery period after salinity was returned to 15 ppt (Figure 2). For the low fluctuation groups (LT and WT), individuals started at a salinity of 15 ppt, then were reduced to 10 ppt an hour later by adding 19 L (5‐gal) of DI water. They were then held at 10 ppt for 4 h before being returned to 15 ppt salinity, replicating the conditions killifish experience in reference areas during tidal events (Figure 2a; Bickley and Anderson 2025). For the high fluctuation groups (LTR and WTR), individuals started at 15 ppt, were reduced to 10 ppt after 1 h, then further reduced to 5 ppt after an additional hour. These individuals were held at 5 ppt for 3 h before being returned to 15 ppt salinity, simulating the conditions killifish more commonly experience in intermediate‐urbanized coastal areas during high tide and terrestrial runoff events (Figure 2b; Bickley and Anderson 2025). At the end of the 7‐h experimental period, all fish were euthanized by performing a cervical spinal transection with sharp dissecting scissors, following the IACUC protocols of Auburn University. Total length and weight of each fish were recorded, and whole gills were collected and preserved in DNA/RNA shield (Zymo) at −80°C, for differential gene expression and analyses of genetic differentiation. Gill tissue from five out of six fish in each tank were randomly selected and retained for subsequent analyses (final sample size: *n* = 20 per population; *n* = 10 per treatment; *n* = 5 per tank).

### 2.3 RNA Extraction, Processing, and Sequencing

Total RNA was extracted from whole right gill samples using the Zymo Quick‐RNA Miniprep kit following the manufacturer's protocol with minor modifications as outlined. Briefly, approximately 50 mg of gill tissue was homogenized in 200 μL DNA/RNA shield. Samples were combined with 15 μL Proteinase K (PK) and 30 μL PK digestion buffer, then incubated overnight at room temperature (~21°C). Following overnight digestion, samples were vortexed at maximum RPM for 2 min, and 300 μL of the supernatant was transferred to a new tube. After supernatant transfer, total RNA was extracted following the manufacturer's protocol. Total RNA was eluted in 60 μL DNase/RNase free water. The concentration of the extracted RNA was quantified using a Qubit 4 Fluorometer (Invitrogen) and its quality was assessed on a 1% agarose gel. Samples with concentrations ≤ 50 ng/μL were purified using the Zymo RNA Clean and Concentrator Kit, following the manufacturer's protocol. Out of the 40 samples collected, a total of 37 (LB = 20; WB = 17) were successfully extracted. Purified total RNA was sent to Novogene Corporation for library preparation and RNA sequencing. Libraries were sequenced on an Illumina NovaSeq6000, generating 150‐bp paired‐end reads.

The quality of the raw reads was checked using FastQC v0.12.1. Low quality sequences and adapters were trimmed using Trimmomatic v0.39 (parameters: ‐phred33 ILLUMINACLIP:adapters.fa:2:35:10 HEADCROP:10 LEADING:10 TRAILING:10 SLIDINGWINDOW:5:30 MINLEN:36; Bolger et al. 2014). The quality of the filtered reads was assessed using FastQC v0.12.1. Reads were then mapped to the published genome of the Atlantic killifish (mummichog), *F. heteroclitus* (publicly available on NCBI at accession number: GCF_011125445.2) using HISAT v2.2.0 (parameters: ‐p 8 ‐k 3 ‐x ‐1 ‐2 ‐@ 8 ‐Sbh>; Kim et al. 2019). A gene expression matrix was generated using StringTie v2.2.1 (parameters: ‐p 8 ‐e ‐B ‐G ‐o; Pertea et al. 2015) for downstream analyses. An overview of the read‐processing pipeline and all associated scripts are available at: https://github.com/kmeaton/RNASeq_Workshop.

### 2.4 Differential Gene Expression

Differential gene expression analyses were conducted using DESeq2 v1.44.0 (Love et al. 2014) in RStudio v4.4.1 (Posit team 2024). The model included population origin, treatment, and their interaction as fixed factors. Tank identity could not be included in the model with the other factors because it would result in a rank‐deficient design matrix. Instead, expression estimates reflect variation among individual samples within each population origin × treatment combination, with independent tanks providing biological replication. A likelihood ratio test was carried out to assess the differential expression across all four groups, to assess the level of divergence associated with origin of population and the amount associated with treatment. We identified differentially expressed genes (DEGs) by selecting those with an adjusted *p*‐value (*p*adj) < 0.05, after applying the Benjamini‐Hochberg correction, which controls the false discovery rate (FDR) and reduces the likelihood of false positives due to multiple comparisons. The gene expression matrix was transformed using the variance stabilizing transformation function and visualized on a principal component analysis (PCA) and sample‐to‐sample heatmap to identify potential outliers. A Wald test was performed to identify DEGs (*p*adj < 0.05) for pairwise comparisons among every combination of site and salinity fluctuation (i.e., LT vs. WT, LTR vs. WTR, LT vs. LTR, WT vs. WTR, LT vs. WTR, and LTR vs. WT).

To detect genes that were consistently expressed or treatment‐induced in one population relative to the other, we analyzed the effect of frontloading or backloading on gene expression (Barshis et al. 2013). For this, we analyzed gene expression data based on population origin to determine the effect of treatment. Backloaded genes are those that become significantly upregulated in response to environmental stress, whereas frontloaded genes are those that have a level of expression comparable to or higher in control samples relative to treatment samples (Collins et al. 2020; Ruggeri et al. 2023). Due to the nature of the study, backloaded genes were identified as those with a *p*adj < 0.05 and log_2_FoldChange > 1 when individuals were exposed to environmental conditions routinely experienced at the other site (i.e., reference population at high fluctuation, LTR, and urban population at low fluctuation, WT). For each set of backloaded genes, normalized expression values were used to compare expression patterns across populations to identify candidate frontloading genes. Home conditions were defined by sub‐setting samples according to their origin and corresponding baseline treatment (i.e., reference at tide, LT; urban at tide/runoff, WTR). To test for potential frontloading, we compared the expression levels of the backloaded genes from one population to the mean expression of those same genes in the home conditions of the other population (i.e., comparing individuals exposed to the tide treatment, WT compared to LT; comparing individuals exposed to the tide/runoff treatment, LTR compared to WTR) and visualized these comparisons on boxplots. We further evaluated the strength of potential frontloading by applying thresholds ≥ 1× (equal to baseline expression), ≥ 1.2× (20% higher), and ≥ 2× (100% higher). Heatmaps were generated to determine whether candidate frontloaded genes were consistently expressed across individuals within each home group.

A Mann–Whitney *U* test was used to identify potentially significantly enriched gene ontology (GO), following the GO‐MWU pipeline provided by Wright et al. (2015). Here, the log_2_FoldChange of the pairwise analyses of differential expression was used to identify the GO categories at the top (upregulated) or bottom (downregulated) of the comparisons. Significant gene categories were identified as those with an FDR of < 0.05. The GO analysis was adapted from https://github.com/z0on/GO_MWU and the complete R scripts for all analyses can be found at: https://github.com/AClark98/KillifishUrbanForested‐RNA.

### 2.5 Weighted Gene Co‐Expression Network Analysis

To identify genes of high correlation related to location and osmotic regulation, we conducted a weighted gene co‐expression network analysis (WGCNA; Langfelder and Horvath 2008). Genes with fewer than 10 reads across all samples were removed prior to downstream analyses. Normalization and variance stabilization of the filtered gene counts were performed using the DESeq2 package (Love et al. 2014) in RStudio (Posit team 2024). A dendrogram was created to visualize potential outliers using a power parameter of 10, selected based on the point of stability in the scale independence and mean connectivity curves. Gene modules were constructed using a signed network topological overlap matrix and merged via hierarchical clustering based on dissimilarity, retaining only modules with a minimum of 30 genes. Correlations (*r*) between modules and traits of interest (i.e., population, treatment, and their interaction) were calculated using the moduleTraitCor function. We applied a linear model to each module to identify those with the most significant differential expression. Genes within modules most significantly correlated with a trait of interest were filtered to identify hub genes, defined as those with high module membership (|MM| > 0.8) and gene significance (|GS| > 0.2), thresholds commonly used to identify genes strongly associated with a given module (Li et al. 2020, 2024). Using the GO‐MWU package in RStudio (https://github.com/z0on/GO_MWU), a Fisher's test was used to identify enriched GO categories among the modules associated with population, treatment, and their interaction.

### 2.6 Analysis of Genetic Differentiation

To evaluate genetic differences among the two populations, transcriptome‐derived single nucleotide polymorphisms (SNPs) were identified using BCFtools v1.13 (Samtools; Danecek et al. 2021). First, variant likelihoods were generated using *bcftools mpileup* with the parameters *‐C 50 ‐q 30 ‐I*, which filters out low‐confidence read alignments, requires a minimum mapping quality score of 30, and ignores indels during likelihood calculation. Variants were then called using *bcftools call ‐m ‐Ov ‐V indels ‐v* to exclude indels from the final output and only keep variant sites across individuals. The resulting variant set was further filtered using vcfutils.pl. varFilter with the options *‐d 50* and *‐‐max‐missing 0.90* to remove sites with low read depth and retain variants present in ≥ 90% of individuals across populations. To reduce linkage disequilibrium, SNPs were pruned using PLINK v1.9 (www.cog‐genomics.org/plink/1.9/; Chang et al. 2015) to only keep sites separated by at least 1 Mb with an R^2^ < 0.2. The final VCF file contained only high‐quality, biallelic SNPs suitable for downstream population structure analyses. We calculated the inbreeding coefficient (FIS; PLINK v1.9), nucleotide diversity (*π*; VCFtools v0.1.16; Danecek et al. 2011), expected heterozygosity (*H*_e_; VCFtools v0.1.16), and observed heterozygosity (*H*_o_; VCFtools v0.1.16) to measure within‐population genetic variation, and the pairwise fixation index (*F*_ST_; VCFtools v0.1.16) to estimate genetic variation between populations. Outlier SNPs were identified using the OutFLANK program (Whitlock and Lotterhos 2015) in RStudio v4.4.1 (Posit team 2024). Population structure was further investigated using ADMIXTURE v1.3.0 (Alexander et al. 2009), running K = 1 through K = 10 with 5 replicates per K. Cross‐validation (CV) errors and log‐likelihood scores were extracted to determine the optimal K and visualized on an admixture graph. All bioinformatic analyses were completed through the Alabama Supercomputer Authority.

## 3 Results

The experiment was successfully conducted with no mortalities. After trimming adapters and removing low‐quality sequences, there was an average of 10,881,259 reads per sample (±1,021,663; SD). Clean reads were mapped to the reference genome of the closely related *F. heteroclitus* (Atlantic killifish; NCBI RefSeq: GCF_011125445.2) with an average mapping rate of 72.46%.

### 3.1 Differential Gene Expression

**FIGURE 3:**
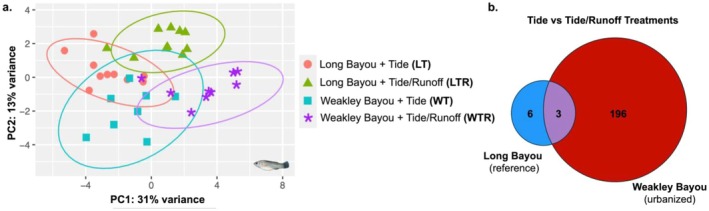
(a) Principal component analysis (PCA) based on 112 differentially expressed genes (DEGs; *p*adj < 0.05) between all four treatment groups: Long Bayou + Tide (LT; *n* = 10), Long Bayou + Tide/Runoff (LTR; *n* = 10), Weakley Bayou + Tide (WT; *n* = 8), and Weakley Bayou + Tide/Runoff (WTR; *n* = 9). (b) Venn diagram showing the number of shared differentially expressed genes for Tide vs. Tide/Runoff treatments between Long Bayou and Weakley Bayou.

The likelihood ratio test across all four groups identified 112 significant DEGs, representing the combined effect of population origin and treatment (58 upregulated and 54 downregulated; Figure 3a). Principal component analysis of the variance‐stabilized expression data showed clear separation by treatment along PC1 and by population origin along PC2, with overlap among tanks within each condition (Figure S1), indicating minimal tank‐associated structure. When the effect of the origin of population was removed, 58 genes (37 upregulated and 21 downregulated) were associated with the effect of treatment exclusively. Additionally, removing the effect of treatment revealed that 27 genes (12 upregulated and 15 downregulated) were associated with population origin exclusively. These findings indicate that the observed gene expression patterns are influenced by a modest non‐additive interaction between the two variables.

The number of significant DEGs, significantly enriched GO terms, and the function of enriched GO terms for each pairwise comparison are presented in Table 1. The largest number of DEGs was found between the two populations exposed to the high‐fluctuating treatment (LTR vs. WTR = 294). Upregulated gene functions in the reference group exposed to high fluctuation (LTR) included immune response (*GIMAP7*, *galactose‐specific lectin nattectin‐like*, and *GIMAP4*; Klangnurak and Tokumoto 2017; Saleh et al. 2019; Rehman et al. 2021), replication fork stability (*DONSON*; Kermi et al. 2019), transcription repression (*GFI1*; Beauchemin and Möröy 2020), ion transport (*MARVELD3*; Steed et al. 2009). For this group, downregulated genes were involved in adult cellular development/differentiation and homeostasis (*NR5A2*; Sandhu et al. 2021), glycosylation (Yamakawa et al. 2018), and wound healing (*Duox1* and *flotillin 1*; de Oliveira et al. 2014; Rios Morris et al. 2017). GO categories that were upregulated in the reference group exposed to high fluctuation (LTR) included “L‐amino‐acid‐oxidase activity,” “DNA replication initiation,” “metallo‐sulfur cluster assembly,” “proton channel activity,” and terms such as “regulation of neuron differentiation,” “anatomical structure morphogenesis,” “dephosphorylation,” “cell–cell adhesion,” and “integrin complex” were downregulated.

**TABLE 1:** Number of differentially expressed genes (DEGs) and gene ontology (GO) categories for each pairwise comparison across the three GO groups: Molecular Function (MF), Biological Processes (BP), and Cellular Components (CC).

Comparison	Direction	DEGs	Significant GO terms			Relevant GO term functions
			MF	BP	CC	
LT vs. WT	Up	4	11	12	10	Ion transmembrane activity, signal transduction, structural integrity of the cell, DNA repair, upregulation of metabolism, upregulation of gene expression
	Down	2	6	21	15	
LT vs. LTR	Up	5	37	38	32	Ion channel activity, ion exchange activity, immune response, hormone stimulus, structural integrity of the cell, enzyme activity, wound repair
	Down	4	20	44	50	
LT vs. WTR	Up	29	14	35	18	Ion transmembrane activity, hormone stimulus, signal transduction, cell apoptosis, enzymatic activity, ATP‐dependent processes
	Down	40	21	20	5	
WT vs. WTR	Up	96	37	84	35	Ion channel activity, signal transduction, enzymatic activity, immune response, anticoagulant, hormone stimulus
	Down	103	26	38	13	
LTR vs. WTR	Up	170	41	91	58	Ion binding, ion transmembrane activity, anticoagulant, structural integrity of the cell, ion channel activity, ATP‐dependent processes
	Down	124	57	49	32	
LTR vs. WT	Up	7	12	23	25	Ion transmembrane activity, enzymatic activity, ion channel activity, ion binding, anticoagulant, positive regulation of metabolism, positive regulation of gene expression, hormone stimulus, cell structural integrity, ion transport
	Down	22	24	23	20	

The comparison of reference vs. urban individuals exposed to the low fluctuating treatment (LT vs. WT) resulted in 6 DEGs. The reference group exposed to low fluctuation, LT, showed upregulation for DNA damage response (*trip12*; Keyan et al. 2023), while osmoregulation was downregulated (*atp1b2b*; Armesto et al. 2015). Individuals from this treatment saw upregulation of GO categories such as “cardiac muscle tissue development,” “cargo receptor activity,” “striated muscle contraction,” “active ion transmembrane transporter activity,” “signaling receptor activity,” and a downregulation in “chromosome segregation,” “cell division,” “cell cycle process,” “nucleobase‐containing compound transport,” and “mitotic cell cycle”.

To understand the potential impact of population origin on osmotic stress response, we compared the gene expression of individuals from each population when exposed to the two fluctuating treatments. As this study included only one urban and one reference population, these population origin comparisons reflect differences between these two specific populations rather than a replicated test of urbanization. The number of DEGs among individuals from the urban site Weakley Bayou when exposed to high and low fluctuations (WT vs. WTR) resulted in 199 DEGs. Surprisingly, this was more than 20 times greater than the number of DEGs for the reference site, Long Bayou, where the comparison of low (LT) vs. high fluctuations (LTR) showed 9 DEGs (Figure 3b). Moreover, these comparisons revealed only three overlapping genes based on population origin (Table S1): *calpain 2* (*CAPN2*; Salem et al. 2005), *serum amyloid p‐component‐like* (*SAP/SAP‐like*; Talbot et al. 2021), and *solute carrier family 25 member 55a* (*slc25a55a*; Garcia‐Reyero et al. 2016). The low‐fluctuation treatment from the urbanized site, WT, showed an upregulation of genes associated with histone modification and DNA damage response (*tdrkh*; Liu et al. 2022), cell cycle arrest (*cdkn3*; Noya et al. 2022), microtubule organization (*tppp2*; Orosz 2015), immunity (*GIMAP7* and serum amyloid p‐component‐like; Klangnurak and Tokumoto 2017; Talbot et al. 2021), and a downregulation in glycosylation (*cmah*; Yamakawa et al. 2018), ion transport (*slco1d1*, *aqp1*, *CLCN2*, and *slc12a10.1*; Popovic et al. 2014; Horng et al. 2015; Root et al. 2021; Ota et al. 2024), neuronal development and regeneration (*ppp3cb*, *adgrg6*, *klf13*, and *sema7a*; Hammond and Udvadia 2010; Baxendale et al. 2021; Ávila‐Mendoza et al. 2024; Dasgupta et al. 2024), hearing loss (*tbc1d24*; Sarosiak et al. 2025), and vascular development (*calcrla*; Nicoli et al. 2008). This comparison also displayed the highest number of GO terms (*n* = 236) with terms such as “negative regulation of mitotic cycle,” “G protein‐coupled receptor activity,” “immune receptor activity” and “haptoglobin‐hemoglobin complex” being upregulated, and “insulin receptor signaling pathway,” “calcium ion transmembrane transport,” “neuron projection guidance,” “cell adhesion,” and “blood microparticle” showing downregulation in WT.

The reference population at low fluctuation, LT, saw an upregulation of genes associated with DNA mismatch repair (*msh5*; Busch and DiRuggiero 2010), immunity (*SAP/SAP‐like*; Talbot et al. 2021), metabolic pathways (*st6galnac1.1*; Hu et al. 2016), muscle development and maintenance (*CAPN2* and *slc2a6*; Salem et al. 2005; Shi et al. 2018) and downregulation of neural crest regeneration (*AEBP2*; Strobl‐Mazzulla et al. 2012), and hormone‐associated activity (*slc25a55a* and *TH/dio1*; Garcia‐Reyero et al. 2016; Walter et al. 2019). Upregulated functional GO categories in LT included “regulation of wound healing,” “active transmembrane transporter activity,” “lipoprotein metabolic process,” “muscle structure development,” and downregulated categories included “oxidoreductase activity,” “response to stress,” “immunoglobin receptor binding,” “nucleic acid transport,” and “anaphase‐promoting complex.”

There were 69 differentially expressed genes between the two populations at their respective baseline treatment: the reference site at low‐fluctuation (LT) and the urbanized site at high‐fluctuation (WTR). Genes involved in immunity (*GIMAP7*, *galactose‐specific lectin nattectin‐like*, and *SAP/SAP‐like*; Klangnurak and Tokumoto 2017; Saleh et al. 2019; Talbot et al. 2021), development and regeneration (*wt1b*, *tubulin alpha‐1C chain*, *nox1*, and *pnhd*; Perner et al. 2007; Ilies et al. 2012; Weaver et al. 2016; Paraiso et al. 2019), neuropathy (*arhgap19*; Dominik et al. 2024), and cytoskeleton integrity (*CAPN2* and *tubulin alpha‐1B chain*; Salem et al. 2005; Eastlake et al. 2017) were upregulated in the LT treatment. Genes involved in antiviral immune regulation (*MX1*, *HERC3*, and *ZNFX1*; Altmann et al. 2004; Paparisto et al. 2018; Jia et al. 2023), transcription regulation (*NACC2*; Pan et al. 2023), glycosylation (*cmah*; Yamakawa et al. 2018), osmoregulation (*trpm4a*, *CLCN2*, *trpv4*, and *slc12a10.1*; Kastenhuber et al. 2013; Root et al. 2021; Hori et al. 2022; Ota et al. 2024) were downregulated in LT. Functional GO categories that were upregulated in LT included “leukocyte mediated cytotoxicity,” “growth factor receptor binding,” “structural constituent of cytoskeleton,” “electron transfer activity,” “oxidoreductase activity,” with GO categories such as “GTPase activator activity,” “GTPase binding,” “transmembrane receptor protein kinase activity,” “enzyme activator activity,” and “bioactive lipid receptor activity” being downregulated.

The comparison of individuals from the reference site exposed to high fluctuation (LTR) to samples from the urban site at low fluctuation (WT) resulted in 29 DEGs. Samples from LTR showed upregulation of gene functions associated with neurological homeostasis (*AE binding protein 2* and *zDHHC16*; Strobl‐Mazzulla et al. 2012; Shi et al. 2015). The LTR samples showed downregulation of genes involved in muscle wasting (*CAPN2*; Salem et al. 2005), neuro‐development and growth (*IGFBP‐6b*, *GAP43*, *ANXA2a*, *nox1*, *ap4s1*, and *eEF1A1*; Wang et al. 2009; Caprara et al. 2014; Saxena et al. 2016; Weaver et al. 2016; D'Amore et al. 2020; Idigo et al. 2020), cell cycle control (*14–3–3γ* and *tfam*; Masters and Fu 2001; Otten et al. 2020), and sensory (*tppp2* and *CNDP1*; Orosz 2015; Schmöhl et al. 2019). Functional GO categories for LTR were upregulated in “phosphatidylserine binding,” “solute:cation symporter activity,” “positive regulation of gene expression,” “protein folding,” “ATPase activity,” “positive regulation of metabolic process,” and downregulated in “intermediate filament,” “chromosome organization involved in meiotic cell cycle,” “anatomical structure morphogenesis,” “system development,” “cell recognition,” and “neuron projection.”

### 3.2 Front‐ and Back‐Loading Analysis

The analyses revealed three genes that were backloaded for the Long Bayou population (LTR) and 14 for the Weakley Bayou population (WT). For Long Bayou, two of the backloaded genes were associated with cytoskeleton integrity (*capn12*; Bochner et al. 2017) and innate immunity (*MPEG1*; Ellett et al. 2011), while the third lacked annotation. Most of the Weakley Bayou backloaded genes were unannotated, with the few annotated genes being involved in DNA damage response (*tdrkh*; Liu et al. 2022) and digestive system maintenance (*dnph1/RCL*; Zhu et al. 2021). Meanwhile, the frontloading test showed that three genes were frontloaded in the urbanized Weakley Bayou population (WTR; Figure S2). Further, 14 genes were frontloaded in the reference Long Bayou population at low salinity fluctuation (LT; Figure S3). When applying a stronger threshold of 20% above baseline expression, no frontloading genes were detected in the Weakley Bayou population, and one gene (unannotated) remained frontloaded in the Long Bayou population. The strongest threshold of double the baseline expression resulted in no frontloading gene candidates for both populations, indicating that while baseline frontloading is present, strong frontloading is absent.

### 3.3 Weighted Gene Co‐Expression Network Analysis

**FIGURE 4:**
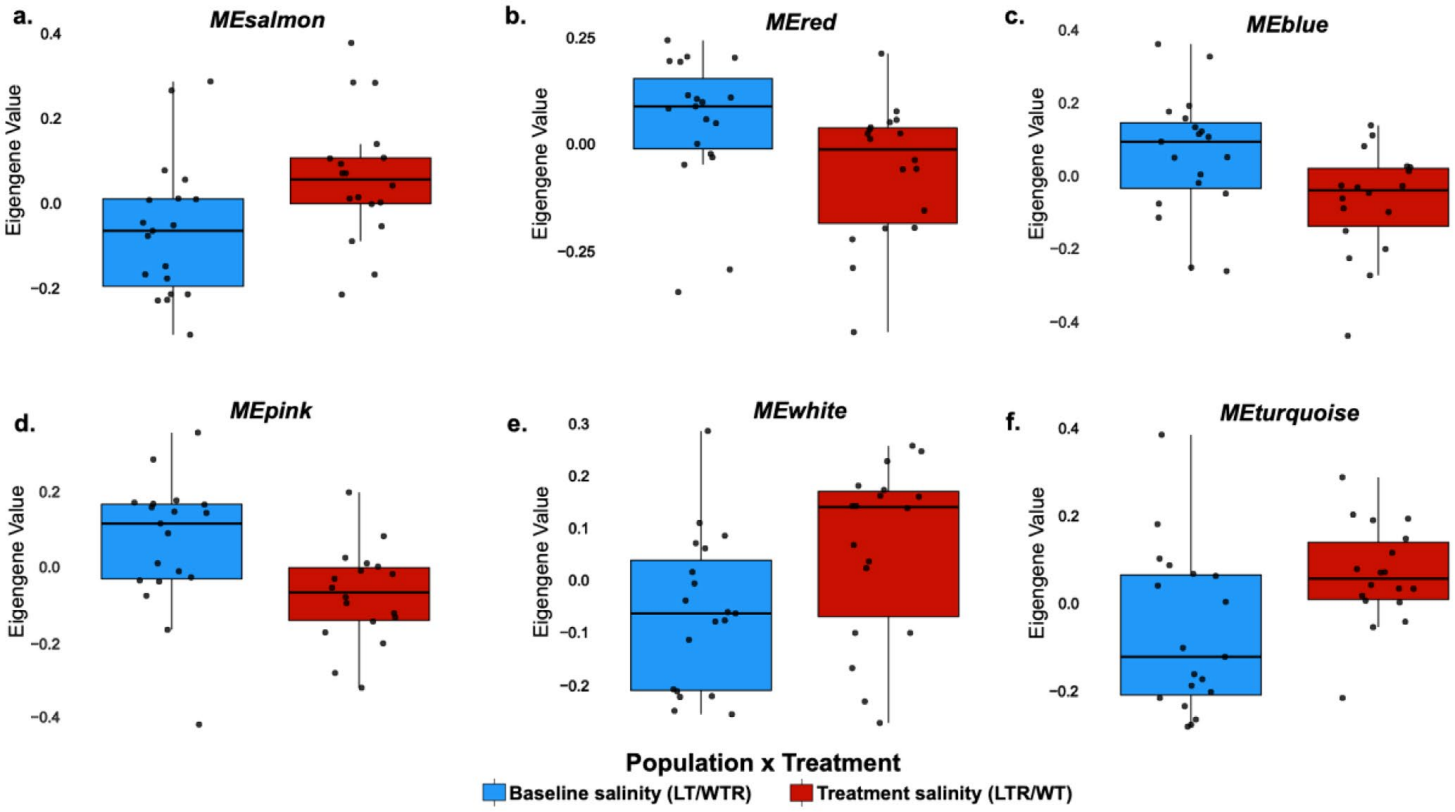
Boxplots of WGCNA modules showing significant correlation with the combination of population and treatment. Baseline individuals are represented in blue (reference site at low fluctuation, LT; and urban site at high fluctuation, WTR; *n* = 19) and treatment individuals are represented in red (reference site at high fluctuation, LTR; urban site at low fluctuation WT; *n* = 18).

Our WGCNA analysis resulted in 24 modules of co‐expressed genes (Figure S4). Two of these modules were significantly (*p* < 0.05) associated with treatment: *MEdarkred* contained 201 genes and *MEdarkgreen* contained 806 genes. Six of these modules were significantly correlated with the interaction between population and treatment: *MEsalmon* contained 353 genes, *MEred* contained 1406 genes, *MEblue* contained 2156 genes, *MEpink* contained 626 genes, *MEwhite* contained 138 genes and *MEturquoise* contained 2668 genes (Figure 4, Figure S5). *MEdarkgreen* was the most strongly correlated module with the treatment trait (*r* = 0.47, *p* = 0.003) and showed increased expression of genes involved in sprouting angiogenesis and hematopoietic stem cell development (*arhgef9b* and *rnf111*; Wakayama et al. 2015; Liu et al. 2024), regeneration (*RERE/atrophin2* and *apc*; Asai et al. 2006; Goessling et al. 2008), immune regulation (*leng8*; Wang et al. 2021), and chromatin modification and segregation (*INO80D*, *kat6a*, *kdm6al*, *BRD3*; Shameer et al. 2014; Sen et al. 2018; Sen et al. 2020; Lee et al. 2023). The Fisher's test indicated enrichment in 12 GO categories including “RNA binding,” “chromatin remodeling,” “transcription coregulator activity,” “BMP signaling pathway,” and “spliceosomal complex.”

Of the six modules associated with the interaction between population origin and treatment, *MEpink* exhibited the strongest correlation (*r* = −0.42, *p* = 0.009; Figure 4, Figures S4 and S5), showing decreased expression in hub genes associated with signal transduction (*sash1a* and *tgfbr3*; Shamay‐Ramot et al. 2015; Wang et al. 2019) and development (*nrp2b*, *raraa*, *LRP1*, and *TENM3*; Yu et al. 2004; Hale et al. 2006; Pi et al. 2012; Cheung et al. 2019). There were nine GO categories overrepresented for the *pink* module including “GTPase activator activity,” “semaphorin receptor activity,” “enzyme regulator activity,” “enzyme activator activity,” and “signaling receptor activity.” The module *MEturquoise* displayed the strongest positive correlation with the interaction between population origin and treatment (*r* = 0.41, *p* = 0.01; Figure 4, Figures S4 and S5) and contained hub genes involved in the regulation of transcription and translation (*gtf2a2*, *LSM3*; Aedo et al. 2019; Sadeghi et al. 2023), metabolism (*scp2b*; Tian et al. 2026), protein protection (*hint1*, *dnaja2a*; Jeffries et al. 2015; Taugbøl et al. 2022), and cellular homeostasis (*nhp2*, *apip*, *RPL5*; Williams et al. 2006; Jayasundara et al. 2013; Wan et al. 2016). There were 86 GO categories enriched for the turquoise module including “structural constituent of ribosome,” “structural molecule activity,” “protein folding,” “cellular response to DNA damage stimulus,” “ribosomal subunit,” and “mitochondrial ribosome.”

### 3.4 Analysis of Genetic Differentiation Through SNPs

**FIGURE 5:**
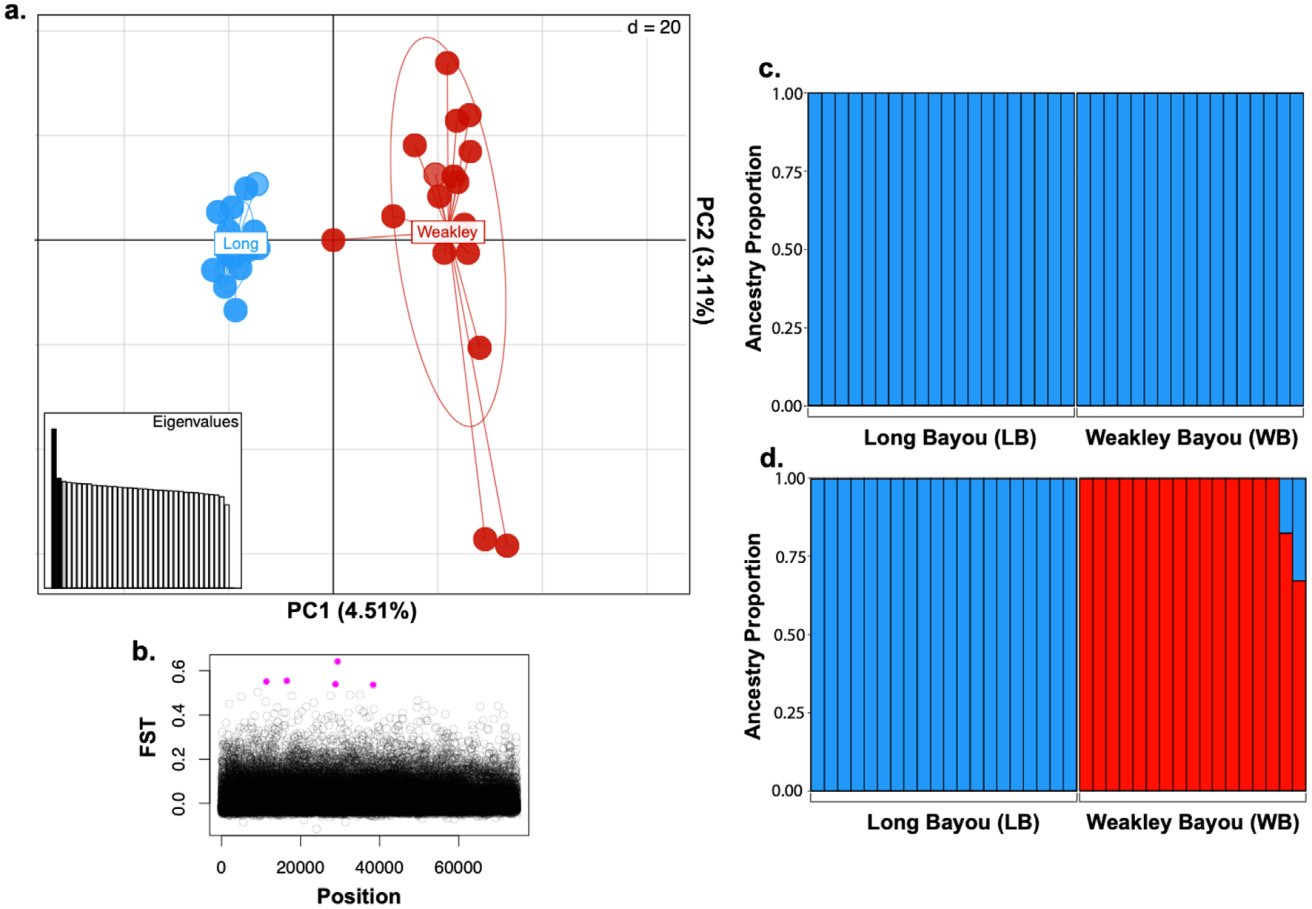
(a) PCA based on SNPs called from RNA‐seq data for individuals from the reference site Long Bayou (blue; *n* = 20) and urbanized Weakley Bayou (red; *n* = 17) populations. (b) *F*_ST_‐heterozygosity outlier analysis. Admixture graphs suggest a K value between (c) 1 and (d) 2.

After filtering and pruning to reduce linkage disequilibrium, our analysis revealed 74,759 high confidence SNPs (*R* ^2^ < 0.2). The FIS revealed that the urbanized site Weakley Bayou was more genetically diverse, indicating higher levels of outbreeding when compared to individuals from the reference Long Bayou population (Figure 5a). Individuals from the Weakley Bayou population also had higher nucleotide diversity, which is consistent with inbreeding coefficient estimates (Table 2). The weighted Weir and Cockerham pairwise *F*_ST_ between the two sites was 0.021 (*p* < 0.05), suggesting small but significant genetic differentiation. OutFLANK detected five high confidence *F*_ST_ outliers, associated with mitotic spindle assembly (*EMAP like 3*; *F*_ST_ = 0.537), proteolysis (*ubiquitin specific peptidase 40*; *F*_ST_ = 0.540), transmembrane ion transport (*solute carrier family 40 member 4*; *F*_ST_ = 0.552), structural integrity (*collagen, type I, alpha 1b*; *F*_ST_ = 0.555), and hemoglobin production and antioxidative protection (*ATP‐binding cassette sub‐family B member 10, mitochondrial*; *F*_ST_ = 0.643; Figure 5b). The admixture analyses were not conclusive, as the CV error was slightly lower for K = 1 (0.538) than for K = 2 (0.586), while log‐likelihood values were higher for K = 2 (−2100148.893) than for K = 1 (−2162703.421; Figure 5c,d).

**TABLE 2:** Summary of population genetic statistics for Long Bayou and Weakley Bayou. Reported values represent the number of individuals, inbreeding coefficient (FIS), nucleotide diversity (*π*), expected heterozygosity (*H*_e_), and observed heterozygosity (*H*_o_) for each population.

Population	# of individuals	FIS	*π*	*H*_e_	*H*_o_
Long Bayou	20	0.019	0.103	0.256	0.386
Weakley Bayou	17	0.004	0.106	0.254	0.385

## 4 Discussion

In this study we evaluated if individuals exposed to greater and more frequent salinity fluctuations in urban sites respond to changes in salinity in the same way as fish from reference sites. The results of the differential expression analysis showed that the largest differences in gill gene expression were found between individuals from the reference site (Long Bayou) and the urban site (Weakley Bayou) exposed to a simulated high‐fluctuating treatment. The results indicate that there are differences in the potential for acclimation among groups previously exposed to high and low fluctuations. We also detected small but significant genetic differences between individuals from low‐fluctuating (reference) and high‐fluctuating (urbanized) sites, with higher genetic diversity observed in individuals from the urbanized site.

### 4.1 Molecular Mechanisms Associated With Osmotic Regulation

Under changing salinity conditions, one of the primary functions of gills is to exchange water and ions for osmotic balance (Evans et al. 2005). However, the results from this study show that the molecular mechanisms that promote osmotic adjustments can differ among populations originating from distinct environmental backgrounds. This is evidenced by the limited overlap in differentially expressed genes between populations from reference and urbanized sites exposed to quick salinity changes in an aquarium setting, where only three genes were shared among the two populations. Exposure to the high‐fluctuating treatment led to the downregulation of *calpain 2* (*CAPN2*), which are calcium‐dependent proteases responsible for regulating a multitude of processes including muscle atrophy and cytoskeletal remodeling (Macqueen et al. 2010). Another gene that was downregulated was *serum amyloid‐component‐like* (*SAP/SAP‐like*), which plays a role in immune defense in bony fish (Lund and Olafsen 1999; Wang and Sun 2016; Aversa‐Marnai et al. 2022; Gao et al. 2025). In contrast, the high‐fluctuating treatment led to the upregulation of the *solute carrier family 25 member 55a* (*slc25a55a*) transcript, a mitochondrial transporter involved in metabolism and cellular energy balance (Garcia‐Reyero et al. 2016), consistent with observations in a low salinity exposure study for mud crabs (Mo et al. 2024). Previous transcriptomic studies on salinity stress have reported enrichment of mitochondrial‐related GO categories, indicating higher energetic demands during osmoregulatory acclimation efforts (Kidder et al. 2006; Whitehead, Galvez, et al. 2011; Brennan et al. 2015). The elevated expression of this transporter and suppression of the cytoskeletal remodeling and immune‐associated genes likely reflects a reallocation of energy toward energetically costly ion transport for osmotic equilibrium under fluctuating salinity (Evans and Kültz 2020).

The broader functional GO categories also revealed population‐specific patterns of osmoregulation, related to their corresponding salinity regime, as only 22 GO terms overlapped between sites. The function of the individual genes when grouped into gene ontology categories was consistently upregulated under each population's' baseline conditions (Long Bayou = Tide; Weakley Bayou = Tide/Runoff) and downregulated under each population's treatment conditions (Long Bayou = Tide/Runoff; Weakley Bayou = Tide). This reciprocal pattern suggests that while similar molecular pathways are involved in osmotic regulation across populations, the direction and magnitude depend on their prior exposure to salinity. In other words, molecular pathways that are transcriptionally activated under baseline conditions for one population become suppressed when those individuals are subjected to a non‐native salinity regime, which is consistent with a genotype by environment interaction. Thus, in Gulf killifish, population‐specific regulation patterns pose constraints and shape the acclimatory responses to acute osmotic stress.

### 4.2 Differences in Acclimation to Changing Environments

The acclimation capacity to salinity changes differed strongly between populations, with individuals from the urbanized population displaying a much broader transcriptional response than individuals from the reference population. This large‐scale difference in gene expression suggests that repeated exposure to environmental variability may promote enhanced plasticity. In contrast, the limited response observed in the reference population may reflect reduced plasticity in gene expression when exposed to salinity regimes outside of their typical environmental range.

Enhanced transcriptional plasticity under variable salinity has been documented in other estuarine fishes. For example, Sacramento splittails regularly exposed to higher saltwater intrusion variability exhibited a substantially greater transcriptomic response (977 DEGs) compared to a population from a more stable environment (215 DEGs; Jeffries et al. 2019). Similar patterns have been seen in Atlantic cod (Larsen et al. 2011), Arabian pupfish (Bonzi et al. 2021), and the marine blue mussel (Yévenes et al. 2025), where populations acclimated to higher or more variable salinity regimes exhibit larger numbers of DEGs following salinity challenge than populations from more stable environments. Collectively, these studies suggest that increased environmental heterogeneity may contribute to greater transcriptional flexibility, which may be a key acclimation response in populations exposed to variable conditions (Meier et al. 2014; Jeffries et al. 2019). In this context, the greater number of DEGs observed in the urban population likely reflects an enhanced transcriptional flexibility relative to the reference population.

This pattern is not exclusive to salinity and has also been observed in systems that experience other forms of environmental fluctuations. For example, higher tolerance to contaminants has been documented in the closely related species *F. heteroclitus* , where populations inhabiting chemically polluted sites show increased tolerance relative to those from cleaner sites (Vogelbein et al. 1990; Black et al. 1998; Elskus et al. 1999; Nacci et al. 1999, 2002, 2010; Meyers and Di Giulio 2002, 2003; Ownby et al. 2002; Whitehead, Galvez, et al. 2011). In ecologically divergent populations of Redband trout, thermal plasticity appeared greater in desert populations, when compared to montane populations (Narum and Campbell 2015). These recurring patterns across taxa suggest that long‐term exposure to environmental pressures can drive population‐specific responses, illustrating the potential role of environmental background in shaping acclimatory capacity.

The results from this study suggest there is a small, conserved transcriptional core response to osmotic stress in the two populations of Gulf killifish evaluated in this study. However, it is also relevant to point out that the duration of the experiment (~7 h) could limit the capacity of the reference population to activate a transcriptional response. A study comparing copepods from hypo‐ and hyper‐salinity environments showed individuals exposed to the former were more resistant to low salinity stress than those inhabiting higher salinity regimes (Hahn et al. 2026). However, after 24 h of exposure, both hypo‐ and hyper‐saline individuals showed similar levels of gene expression, and broader overlap in molecular processes. Similar results were seen in the Arabian pupfish, where the number of differentially expressed genes in gills of near‐freshwater fish exposed to saltwater was modest at first, but increased substantially after weeks of exposure (Bonzi et al. 2021). Thus, it remains to be determined if the responses of fish in reference sites remain the same after long‐term exposure (i.e., days and weeks), and if the key difference between both populations is the time it takes to engage the transcriptional plasticity.

Finally, while salinity was the main environmental variable investigated in this study, coastal organisms are often exposed to multiple interacting factors that can also shape acclimatory responses. Variables such as diet, competition, food availability, and additional stressors associated with urbanization (e.g., temperature, contamination, or hypoxia; Freeman et al. 2019) were not evaluated in this study and could have contributed to the observed differences between populations.

### 4.3 Osmoregulation in Urbanized Sites

Within just 7 h of salinity challenge, individuals from the urban population (Weakley Bayou) mounted a broad transcriptional response, demonstrating rapid plasticity in response to osmotic disturbance. Osmotic pressure can immediately trigger a cellular stress response, a highly variable mechanism influenced by the magnitude and direction of salinity change, which can then drive downstream physiological changes (Evans and Kültz 2020). Genes associated with ion transport and homeostasis regulation were differentially expressed and found in enriched GO categories suggesting rapid modification of transmembrane ion movement for intracellular osmotic balance. Acute shifts in salinity can disrupt the ionic gradients in gill epithelial tissues by driving rapid water movement, resulting in cellular swelling or shrinkage (Strange 2004; Kültz 2015). Activation of ion transporters and associated regulator processes likely promotes restoration of cell volume equilibrium under fluctuating salinity conditions.

In addition to the facilitation of ion processes, exposure of the urban population to a salinity treatment different from that experienced in their native environment was associated with the upregulation of functions involved in the negative regulation of the cell cycle, which is consistent with cell cycle arrest. Temporary suppression of cell proliferation during osmotic challenge has been previously documented in green sturgeon (Sardella and Kültz 2014), Delta smelt (Kammerer et al. 2016), and other fishes undergoing salinity transitions and is said to promote remodeling in gill osmoregulatory epithelia to reverse the direction of active sodium and chloride transport across the membrane (Evans and Kültz 2020). Ionocytes that are paused in the cell cycle or targeted for programmed cellular death may subsequently be replaced with ionocytes that are better suited for withstanding the present osmotic pressure (Conte and Lin 1967; Laurent and Dunel 1980; Chretien and Pisam 1986; Evans 2008; W. Marshall 2013; Kültz 2015). The urban population also exhibited a strong representation of pathways associated with energy production or reallocation, metabolic processes, and biosynthesis, which highlights the substantial energetic demands associated with active osmoregulation (Hwang et al. 2011). The pronounced upregulation in cell cycle inhibitor pathways observed in the present study may therefore represent a physiological adjustment to redirect cellular resources toward the restructuring of ionocytes for intracellular stabilization during acute osmotic stress.

Categories related to cytoskeletal organization, intracellular transport, and cell communication were prominently represented, indicating structural and signaling adjustments that maintain cellular integrity during osmotic disturbance. However, even during rapid modification of ionocytes and structural components, acute salinity shifts can induce cellular stress through osmotic imbalance and oxidative damage (Fiol and Kültz 2007; Rivera‐Ingraham et al. 2016). The enrichment of oxidoreductase activity, stress response, and immune‐related pathways indicates that protective mechanisms are in place to counteract cellular damage that persists despite these structural and ionoregulatory adjustments (Dowd et al. 2010; Whitehead et al. 2013). Collectively, the coordinated modulation of multiple physiological adjustments reflects how individuals in the urban population maintain homeostasis during abrupt and unpredictable salinity fluctuations, such as those experienced in coastal systems undergoing development.

### 4.4 Genetic Divergence

The population‐specific gene expression patterns observed in this study are consistent with genotype‐by‐environment interactions (Comstock and Moll 1963) and support the idea of functional divergence between populations. This is partially supported by the results of SNP loci obtained from the transcriptome, which revealed significant, albeit small, genetic differences between the two populations (*F*_ST_ = 0.021; *p* < 0.05). These findings are consistent with previous work examining mitochondrial DNA (mtDNA; COI and Control Region) among urban and reference sites, where both distance and habitat type led to significant genetic differences (Waldo et al. 2023). Our results are also consistent with population structure analyses of *F. grandis* using microsatellite markers, which demonstrated a significant positive relationship between the genetic structure of a population and geographic distance (Williams et al. 2008). This pattern of isolation by distance indicates limited gene flow between populations, particularly between more distant sites (Williams et al. 2008; Oleksiak 2010; Whitehead, Galvez, et al. 2011), which has the potential to promote local adaptation in Gulf killifish populations.

Similar patterns have been observed in the closely related *F. heteroclitus* , where populations exposed to different salinity regimes display both transcriptional and genetic divergence, including nuclear variation detected using amplified fragment length polymorphisms (AFLPs) and differences in mitochondrial haplotypes (e.g., COII and ND5; Whitehead, Galvez, et al. 2011; Whitehead, Roach, et al. 2011). Whole‐genome analyses have identified loci under selection associated with osmoregulatory and stress‐response pathways (Brennan et al. 2018), demonstrating that populations differ at both the genetic and transcriptional levels. It is important to note that population estimates in the present study may be influenced by the limited genetic divergence captured by transcriptome‐derived SNPs, as whole‐genome approaches are still pending in the Gulf killifish. While this limitation could lead us to underestimate the genetic divergence among urban and reference sites, the consistency of our results with prior studies suggests that osmoregulatory responses in the Gulf killifish should be attributed to both natural selection and phenotypic plasticity.

Another salient finding from the population genetic comparisons was that samples from urbanized sites exhibited slightly higher genetic diversity compared to reference sites. This was also observed in Gulf killifish using mtDNA, where urbanized sites harbored a greater number of haplotypes and higher haplotype diversity (Waldo et al. 2023). Interestingly, this pattern of elevated genetic diversity in urban or highly connected sites has been documented in other species, such as the western black widow spider, where urban hubs of connectivity facilitated gene flow and increased genetic variation (Miles et al. 2018). Likewise, transport via vehicles and bait abandonment resulted in higher genetic diversity in the non‐native earthworm *Dendrobaena octaedra* in northern Alberta (Cameron et al. 2007). These examples suggest that urbanized environments may provide conditions that enhance genetic diversity through increased migration, connectivity among subpopulations, or larger effective population sizes (Holderegger and Giulio 2010; Crispo et al. 2011).

### 4.5 Implications for the Future

Fish communities in reference and urbanized tidal creek systems of the GoM are highly susceptible to salinity fluctuations, and the Gulf killifish is a common species persisting across sites and seasons in our study area (Wedge and Anderson 2017). Our findings provide a potential mechanistic explanation for this persistence, where repeated exposure to salinity variability in urban systems may shape regulatory responses, enhancing molecular flexibility to osmotic fluctuations.

Although our data suggest greater plasticity in individuals from the urbanized site, we did not measure fitness‐associated outcomes such as survival, growth, or reproductive success. In some systems, increased plasticity may buffer populations against environmental fluctuations (Chevin et al. 2010; Donelson et al. 2019), whereas in others, local adaptation can reduce plasticity by canalizing responses to anticipating changes (Pigliucci et al. 2006; Ghalambor et al. 2007; Lande 2009; McCairns and Bernatchez 2010). In their review of three urban creeks (including WB) and three reference creeks (including LB), Wedge et al. (2015) found that *F. grandis* collected at urban creeks had lower liver somatic index and caloric density than specimens collected in reference creeks. Future studies that integrate fitness metrics will be critical to understanding whether observed plasticity is representative of an adaptive strategy or a short‐term compensatory response with potential trade‐offs.

As urban growth and salinity variability are expected to expand and increase in coming years, even highly tolerant species such as killifish may be pushed toward their physiological limits. Salinities above 5 ppt are necessary for normal physiological function in the Gulf killifish, while long‐term exposure to ≤ 0.5 ppt can lead to significant impairment (Patterson et al. 2012). This is particularly relevant given that salinity monitoring in the northern GoM has already documented tidal creek salinities in urbanized sites reaching below 5 ppt (Bickley and Anderson 2025). Investigating long‐term responses to osmotic stress near these critical thresholds will be necessary to better predict population persistence under environmental fluctuations. A broader spatial framework should also be considered, incorporating multiple reference and urbanized sites to determine whether observed population differences are driven by urbanization or other site‐specific factors (D. J. Marshall 2024). Such an approach would also help disentangle responses driven by plasticity from those reflecting local adaptation.

## 5 Conclusion

The substantial transcriptional differences between individuals from Long Bayou and Weakley Bayou highlight how even fine‐scale spatial variation (< 20 km) can shape environmental conditions, acclimatory capacity, and stress responses in coastal fishes. Gulf killifish from the urbanized site exhibited pronounced transcriptomic remodeling in response to osmotic changes, while individuals from the reference site showed limited transcriptional adjustments. The main adjustments from the urban population were associated with activation of processes such as ion transport, energy reallocation, negative regulation of cell cycle, and cellular integrity. The results also showed significant population divergence, with potential local adaptation for genes associated with ion transport, structural integrity, immunity, and antioxidative protection, which largely correspond to genes that were differentially expressed. Overall, this study exemplifies how adaptation and plasticity can operate in concert to regulate the response of coastal fishes to environmental fluctuations, and how populations from different environmental backgrounds differ in their molecular responses to abiotic oscillations.

## Author Contributions

**Antrelle D. Clark:** data curation (equal), formal analysis (lead), funding acquisition (supporting), investigation (equal), project administration (lead), validation (supporting), visualization (equal), writing – original draft (lead), writing – review and editing (lead). **Adam Hallaj:** conceptualization (supporting), methodology (supporting), writing – review and editing (supporting). **Christopher J. Anderson:** conceptualization (equal), resources (supporting), writing – review and editing (supporting). **Moisés A. Bernal:** conceptualization (supporting), funding acquisition (lead), methodology (supporting), supervision (lead), validation (lead), writing – original draft (supporting), writing – review and editing (supporting).

## Funding

This work was supported by Auburn University College of Sciences and Mathematics; National Science Foundation, 1831075, 1922697, 1937964.

## Conflicts of Interest

The authors declare no conflicts of interest.

## Supporting information


**Table S1:** Number of shared DEGs between pairwise comparisons below the diagonal and number of shared GO terms between pairwise comparisons above the diagonal.
**Figure S1:** Principal component analysis (PCA) based on 112 differentially expressed genes (DEGS; *p*adj < 0.05) from the two populations (Long Bayou and Weakley Bayou) and the two treatments (Low fluctuation [Tide] and High fluctuation [Tide/Runoff]). Each point represents an individual fish, point colors represent population, point shapes represent treatment, and samples are labeled by tank number to assess potential tank effects.
**Figure S2:** Heatmap of frontloaded genes at ≥ 1× in the Weakley Bayou (WB) population when subjected to its baseline treatment, tide/runoff (WTR). Each column along the *x*‐axis represents an individual fish sample, with the samples corresponding to WTR being labeled as such. Each row along the *y*‐axis represents an individual gene that was identified as a frontloading gene candidate in the WB population. A blue coloration corresponds to a decrease in gene expression, a closer to white coloration corresponds to little to no change in expression, and red corresponds to an increase in expression.
**Figure S3:** Heatmap of frontloaded genes at ≥ 1× in the Long Bayou (LB) population when subjected to its baseline treatment, tide (LT). Each column along the *x*‐axis represents an individual fish sample, with the samples corresponding to LT being labeled as such. Each row along the *y*‐axis represents an individual gene that was identified as a frontloading gene candidate in the LB population. A blue coloration corresponds to a decrease in gene expression, a closer to white coloration corresponds to little to no change in expression, and red corresponds to an increase in expression.
**Figure S4:** Module‐trait correlation heatmap from weighted co‐expression network analysis (WGCNA). The *x*‐axis represents traits of interest (i.e., population, population × treatment, and treatment), and the *y*‐axis shows WGCNA modules with the number of genes per module in parentheses. Each cell displays a correlation value (*r*) between a module and a trait, with module names showing significant correlation to at least one trait of interest in bold and stars indicating significant correlation.
**Figure S5:** Boxplots of WGCNA modules showing significant correlation with the population × treatment trait, displayed by experimental group. Long Tide individuals under baseline conditions are represented in peach (reference site at low fluctuation, LT), Long Tide/Runoff individuals under treatment conditions are represented in green (reference site at high fluctuation, LTR), Weakley Tide individuals under treatment conditions are represented in blue (urban site at low fluctuation, WT), and Weakley Tide/Runoff individuals under baseline conditions are represented in purple (urban site at high fluctuation, WTR). Stars indicate the baseline condition for each population.

## Data Availability

All scripts associated with this study can be found on GitHub using the provided link: https://github.com/AClark98/KillifishUrbanForested‐RNA. Raw RNA‐seq experimental reads may be found under NCBI BioProject #PRJNA1445231. The complete list of differentially expressed genes has been deposited in Figshare (doi:10.6084/m9.figshare.32653179). The complete list of significant Gene Ontology terms has been deposited in Figshare (doi:10.6084/m9.figshare.32653248). The complete list of enriched GO terms for each significant module‐trait relationship has also been deposited in Figshare (doi:10.6084/m9.figshare.32690607).
