# Supplementary material for: Neighboring Gulf Killifish (Fundulus grandis) Populations Differ in Their Plastic and Genetic Responses to Salinity Fluctuations
Source: Ecol Evol. 2026 Aug 7;16(8):e74115. doi: 10.1002/ece3.74115 (PMC13451163; doi:10.1002/ece3.74115)
Supplement: Supplementary file 1 [file ECE3-16-e74115-s001.docx]

**Neighboring Gulf killifish (*Fundulus grandis*) populations differ in their plastic and genetic responses to salinity fluctuations**

Antrelle D. Clark**^1,*^**, Adam Hallaj**^1^**, Christopher J. Anderson**^2^**, and Moisés A. Bernal**^1,3,*^**

Department of Biological Sciences, Auburn University, Auburn, Alabama, USA**^1^**

College of Forestry, Wildlife and Environment, Auburn University, Auburn, Alabama, USA**^2^**

Smithsonian Tropical Research Institute, Balboa, Panama, Republic of Panama**^3^**

*Corresponding authors: Antrelle D. Clark (adc0064@auburn.edu) | Moisés A. Bernal (mab0205@auburn.edu)

Keywords: gill, coastal urbanization, osmotic stress, killifish, environmental fluctuations, salinity

**SUPPLEMENTAL TABLE(S)**

**Supplemental Table 1.** Number of shared DEGs between pairwise comparisons below the diagonal and number of shared GO terms between pairwise comparisons above the diagonal.

|  | LT vs WT | LT vs LTR | LT vs WTR | WT vs WTR | LTR vs WTR | LTR vs WT |
| --- | --- | --- | --- | --- | --- | --- |
| LT vs WT | - | 39 | 23 | 49 | 39 | 19 |
| LT vs LTR | 0 | - | 40 | 69 | 145 | 83 |
| LT vs WTR | 0 | 4 | - | 77 | 81 | 22 |
| WT vs WTR | 3 | 3 | 24 | - | 141 | 43 |
| LTR vs WTR | 0 | 1 | 14 | 30 | - | 73 |
| LTR vs WT | 2 | 3 | 1 | 9 | 4 | - |

**SUPPLEMENTAL FIGURE(S)**

**Supplemental Figure 1.** Principal component analysis (PCA) based on 112 differentially expressed genes (DEGS; padj < 0.05) from the two populations (Long Bayou and Weakley Bayou) and the two treatments (Low fluctuation [Tide] and High fluctuation [Tide/Runoff]). Each point represents an individual fish, point colors represent population, point shapes represent treatment, and samples are labeled by tank number to assess potential tank effects.

**Supplemental Figure 2.** Heatmap of frontloaded genes at ≥1x in the Weakley Bayou (WB) population when subjected to its baseline treatment, tide/runoff (WTR) . Each column along the x-axis represents an individual fish sample, with the samples corresponding to WTR being labeled as such. Each row along the y-axis represents an individual gene that was identified as a frontloading gene candidate in the WB population. A blue coloration corresponds to a decrease in gene expression, a closer to white coloration corresponds to little to no change in expression, and red corresponds to an increase in expression.

**Supplemental Figure 3.** Heatmap of frontloaded genes at ≥1x in the Long Bayou (LB) population when subjected to its baseline treatment, tide (LT) . Each column along the x-axis represents an individual fish sample, with the samples corresponding to LT being labeled as such. Each row along the y-axis represents an individual gene that was identified as a frontloading gene candidate in the LB population. A blue coloration corresponds to a decrease in gene expression, a closer to white coloration corresponds to little to no change in expression, and red corresponds to an increase in expression.

**Supplemental Figure 4.** Module-trait correlation heatmap from weighted co-expression network analysis (WGCNA). The x-axis represents traits of interest (i.e., population, population x treatment, and treatment), and the y-axis shows WGCNA modules with the number of genes per module in parentheses. Each cell displays a correlation value between a module and a trait, with module names showing significant correlation to at least one trait of interest in bold and stars indicating the specific trait(s) with significant correlation.

**Supplemental Figure 5.** Boxplots of WGCNA modules showing significant correlation with the population x treatment trait, displayed by experimental group. Long Tide individuals under baseline conditions are represented in peach (reference site at low fluctuation, LT), Long Tide/Runoff individuals under treatment conditions are represented in green (reference site at high fluctuation, LTR), Weakley Tide individuals under treatment conditions are represented in blue (urban site at low fluctuation, WT), and Weakley Tide/Runoff individuals under baseline conditions are represented in purple (urban site at high fluctuation, WTR). Stars indicate the baseline condition for each population.
